# A Novel Radiomics-Based Tumor Volume Segmentation Algorithm for Lung Tumors in FDG-PET/CT after 3D Motion Correction—A Technical Feasibility and Stability Study

**DOI:** 10.3390/diagnostics12030576

**Published:** 2022-02-23

**Authors:** Lena Bundschuh, Vesna Prokic, Matthias Guckenberger, Stephanie Tanadini-Lang, Markus Essler, Ralph A. Bundschuh

**Affiliations:** 1 Department of Nuclear Medicine, University Hospital Bonn, 53127 Bonn, Germany; markus.essler@ukbonn.de (M.E.); ralph.bundschuh@ukbonn.de (R.A.B.); 2 Department of Physics, University Koblenz-Landau, 55118 Koblenz, Germany; prokic@hs-koblenz.de; 3 RheinAhrCampus, University of Applied Science, 56075 Koblenz, Germany; 4 Department of Radiation Oncology, University Hospital Zurich, University of Zurich, 8091 Zurich, Switzerland; matthias.guckenberger@usz.ch (M.G.); stephanie.tanadini-lang@usz.ch (S.T.-L.)

**Keywords:** lung cancer, positron emission tomography (PET), radiomics, tumor volume segmentation, textural features, radiation therapy treatment planning

## Abstract

Positron emission tomography (PET) provides important additional information when applied in radiation therapy treatment planning. However, the optimal way to define tumors in PET images is still undetermined. As radiomics features are gaining more and more importance in PET image interpretation as well, we aimed to use textural features for an optimal differentiation between tumoral tissue and surrounding tissue to segment-target lesions based on three textural parameters found to be suitable in previous analysis (Kurtosis, Local Entropy and Long Zone Emphasis). Intended for use in radiation therapy planning, this algorithm was combined with a previously described motion-correction algorithm and validated in phantom data. In addition, feasibility was shown in five patients. The algorithms provided sufficient results for phantom and patient data. The stability of the results was analyzed in 20 consecutive measurements of phantom data. Results for textural feature-based algorithms were slightly worse than those of the threshold-based reference algorithm (mean standard deviation 1.2%—compared to 4.2% to 8.6%) However, the Entropy-based algorithm came the closest to the real volume of the phantom sphere of 6 ccm with a mean measured volume of 26.5 ccm. The threshold-based algorithm found a mean volume of 25.0 ccm. In conclusion, we showed a novel, radiomics-based tumor segmentation algorithm in FDG-PET with promising results in phantom studies concerning recovered lesion volume and reasonable results in stability in consecutive measurements. Segmentation based on Entropy was the most precise in comparison with sphere volume but showed the worst stability in consecutive measurements. Despite these promising results, further studies with larger patient cohorts and histopathological standards need to be performed for further validation of the presented algorithms and their applicability in clinical routines. In addition, their application in other tumor entities needs to be studied.

## 1. Introduction

Integration of positron emission tomography (PET) in radiation treatment planning became an essential part for many tumor entities, such as head and neck cancer [1], brain tumors [2] and many more [3]. In particular, in the case of non-small-cell lung cancer (NSCLC), advantages of PET using [^18^F]F-fluorodeoxyglucose (FDG) have been shown, as well as for the delineation of primary tumors [4] and in determining which lymph nodes should be included in the treatment field [5].

Besides the many reported advantages of using PET in treatment planning [3,6], the method of how to delineate the clinical target volume based on FDG-PET images is still an open question and the focus of many discussions [7,8]. While modern treatment systems can deliver doses within a submillimeter of spatial error [9], the error made in delineation may be much larger. Although manual delineation is still considered fine as long as standardization in image viewing is kept at a high level, semiautomatic or even automatic algorithms may be preferred. In general, they show a lower variability but are also faster in processing the images. Several methods for such automatic or semiautomatic segmentation of PET images have been proposed. Methods based on fixed uptake values have been shown to be too inaccurate as these values vary strongly between different patients. The variable threshold, based on the maximum uptake in the lesion itself, was proposed and seemed to give better results [10], especially when including background activity [11]. Gradient-based algorithms have been suggested, as they may behave more similarly to a human reviewer, who will look for changes in intensity rather than for the absolute intensity itself, and have been applied successfully to PET data [12,13]. Recently, a high number of more sophisticated algorithms have been presented, including fuzzy logic [14] and deep learning [15].

At the same time, textural features became more and more important in the context of PET image analysis [16,17,18]. In recent years, textural features representing tumor heterogeneity have been shown to be able to predict the treatment outcome of various tumor entities and various kinds of treatment. Positive results have been reported for malignant melanoma treated with immunotherapy [19] or neoadjuvant radiochemotherapy in colorectal cancer [20]. In the context of stereotactic body radiotherapy, textural parameters have been found to predict the risk of local recurrence and disease-specific overall survival [21]. In combination with machine learning approaches, textural features can also be used to classify PET uptake as pathological or physiological with high precision [22] as well as for therapy response prediction at a high level [23]. Therefore, we assumed textural features to be an optimal base for tumor volume delineation as well. In a preliminary study it was shown that textural features can differentiate between lung tumor tissue and healthy lung tissue with high precision. In addition, some of the textural features showed good stability against PET acquisition time, as shown in a study by Jouanjan et al. in multiple-time-point PET examinations [24].

Although textural features have been used for segmentation purposes in medical imaging before [25,26], to our knowledge the only study about their application in PET was published by Markel and colleagues in 2013 [27]. In this study, textural features were reported as a promising tool for differentiation between lung carcinoma and healthy tissue in PET and CT data as well.

In addition, in the case of lung tumors, especially in the lower part of the lung, respiratory motion can lead to severe artifacts in PET imaging. This can result in relevant errors in tumor volume segmentation [28,29]. In particular, in the context of radiation therapy planning, such artifacts may by of high relevance and can lead to overestimation of the target volume. To overcome this issue, several methods have been proposed, starting with respiratory gating [30] over different methods of motion correction [31,32,33]. Recently, a method based on 4D-CT data, which are also often acquired in the context of radiation therapy treatment planning for lung tumors, was presented with good results [34].

The aim of this study was to develop a new segmentation algorithm based on textural features to improve PET target volume delineation in lung cancer patients based on the previously presented ability of some textural features to differentiate between tumoral tissue and healthy lung tissue. To reduce the effect of respiratory motion, the new algorithm was applied to data corrected for motion with a method described previously [34].

## 2. Materials and Methods

### 2.1. Segmentation Algorithm

All segmentation was performed in in-house-developed software (IDL, Version 8.5, Harris Corporation, Broomfield, CO, USA). For comparison purposes, a standard segmentation algorithm based on a variable threshold of 40% of the maximum uptake in a lesion was implemented. This method is one of the most widely applied algorithms for PET segmentation in the case of NSCLC [35].

For radiomics-based tumor volume segmentation, three textural parameters, Kurtosis (KU), Local Entropy (LE) and Long-Zone Emphasis (LZE), were included. These parameters have been found to be able to distinguish tumoral tissue from normal lung tissue in a previous study [24]. For calculation of the parameter, the following equations were implemented:

$$\mathrm{KU}=\frac{1}{N}\sum_{i=1}^{N}(\frac{x_{i}-X}{stdv})$$

$$\mathrm{LE}=\sum_{i=1}^{M}\sum_{j=1}^{N}{M1}_{ij}\cdot \mathrm{lg}({M1}_{ij})$$

$$\mathrm{LZE}=\frac{\sum_{i=1}^{M}\sum_{j=1}^{N}{M4}_{ij}\cdot j^{2}}{\sum_{i=1}^{N}\sum_{j=1}^{M}{M4}_{ij}}$$

where *n* is the number of voxels of the volume for which the parameter needs to be calculated, *x_i_* is the i-th voxel of this volume, *X* is the mean voxel value in this volume, *stdv* is the standard deviation within this volume, *M*1 is the co-occurrence matrix, and *M*4 is the gray–level–size–zone matrix or intensity size zone matrix of this volume as defined, e.g., in [36,37].

**Figure 1:**
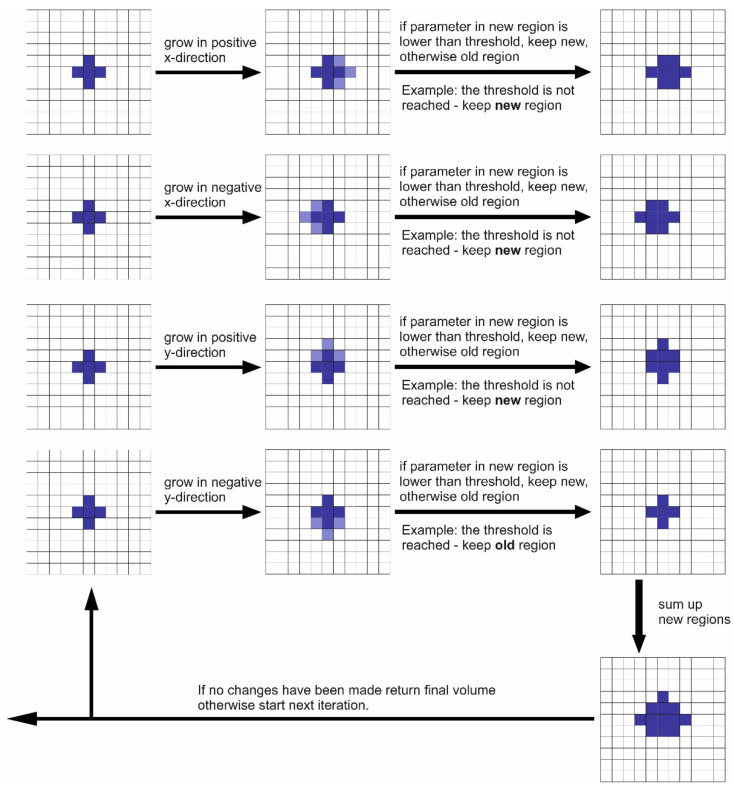
Scheme of the segmentation algorithms.

The proposed segmentation algorithm is based on a region grow algorithm. The basic idea is that the growing of the region is ongoing till the stop criterion is reached. This stop criterion is defined as a threshold of the parameters ZP, LZE and LE. The threshold used for the three textural parameters is based on previous findings [24]. After each region grow step, the parameter is calculated in the new region. For a start volume, 27 voxels are used, defined around a point which is defined by manual interaction of the user (by clicking the center of the lesion to be segmented). The size of 27 voxel as a start volume was tested empirically as the best option. Smaller volumes do not allow adequate estimation of the textural parameters within the volume. If the threshold in the initial volume is not reached, then the first iteration step starts. Based on the start volume in each direction as the region’s grow step of one voxel, starting from the existing voxels in the volume, is performed separately in positive and negative directions. This is illustrated in Figure 1 in two dimensions. In three dimensions, this means that for each iteration step there are 6 separate region grow steps (positive x-direction, negative x-direction, positive y-direction, negative y-direction, positive z-direction, negative z-direction). After each region grow step, the textural parameter is assessed in the new volume. If this value is still lower the threshold, the new volume is accepted; otherwise, the old volume is kept. Consequently, all the volumes for each of the 6 regions grow steps are summed up to end up with the finale volume of this first iteration step (s. also illustration Figure 1). If no changes have been made in any of the 6 region grow steps, the volume is presented as the final segmented volume; otherwise, the next iteration step is started with the new volume as the start volume. The final segmented volume is presented for visual control; in addition, the volume is presented as well as the maximal diameter of the lesion.

#### 2.1.1. Phantom Study

For phantom studies, a commercial extended CT-motion phantom was used (Anzai Medical Co, Ltd., Tokoy, Japan). Three fillable plastic spheres were attached to this phantom, as already reported and shown previously [34]. The volume of the spheres was 3 ccm, 12 ccm and 26 ccm, respectively. In the experimental setting the spheres were filled with watery FDG solution with an activity concentration of 1.2 MBq/ccm. During data acquisition the phantom was set to move with a maximal amplitude of 15 mm and a frequency of 12 per minute.

#### 2.1.2. Patient Study

Data of 5 patients (mean age 69 years, range 56–79 years) with biopsy-proven early-stage NSCLC were used for feasibility analysis of the algorithm. All patients were scheduled for FDG-PET/CT for initial staging and consecutive treatment planning. None of the patients had previous surgery or radiation treatment of the lung. All patients gave written and informed consent for all imaging procedures and agreed to the use of their data in a retrospective evaluation on an anonymized level.

All procedures performed in studies involving human participants were in accordance with the ethical standards of the institutional and/or national research committee and with the 1964 Helsinki Declaration and its later amendments or comparable ethical standards. Due to the retrospective character of the data analysis, an ethical statement was waived by the institutional review board.

#### 2.1.3. Imaging

PET data were acquired for 4 min per bed position (in the case of phantom data for just one bed position, 8–10 bed positions in case of patient studies, depending on the size of the patient) and reconstructed into a 128 × 128 matrix with 5 mm slice thickness using an iterative reconstruction algorithm (4 iterations, 8 subsets). A 5 mm Gaussian post-reconstruction filter was applied to the images for smoothing. Patients fasted for at least 6 h prior the intravenous injection of 301–365 MBq of FDG depending on the body weight. Plasma blood glucose level before injection was between 86 and 121 mg/dL. In patient cases, PET acquisition started between 61 and 76 min after the injection. In phantom studies as well as in patient studies, a low-dose CT was acquired over the PET imaging area for attenuation and scatter correction of PET data. In addition, a 4D-CT was acquired centered on the primary lesion in the context of radiation therapy treatment planning. Based on this information, motion correction was applied as described in [34]. In patient scans, the images not corrected for motion were also evaluated for comparison with the motion-corrected data.

#### 2.1.4. Statistical Analysis

For the phantom data, 20 consecutive segmentation measurements were performed. For results, mean, range and absolute and relative standard deviation are reported. In addition, box-and-whisker plots were performed. Results of feasibility for patient data are reported descriptively. All statistical analysis as well as generation of the plots was carried out in MedCalc (version 12.3.0.0, MedCalc Mariakerke, Ostend, Belgium).

## 3. Results

### 3.1. Phantom Measurements

All four segmentation algorithms worked well in segmentation of the phantom data. When performing 20 consecutive segmentation measurements using the four different segmentation methods, we found a mean volume for the large sphere of 25.0 ccm, 10.8 ccm for the medium sphere and 2.3 ccm for the small sphere using the threshold-based method. When using the method based on Kurtosis, we found a mean volume of 27.0 ccm for the large sphere, 13.5 ccm for the medium sphere and 2.3 ccm for the small sphere.

**Figure 2:**
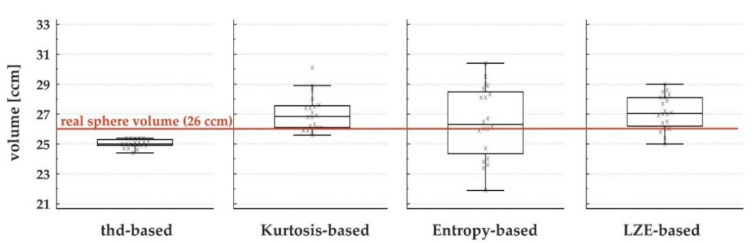
Box-and-whisker plots of the segmentation of the large sphere using the four different algorithms.

The mean volume estimated with Entropy-based measurements was 26.5 ccm for the large sphere, 12.2 ccm for the medium sphere and 3.1 ccm for the small sphere. For the LZE-based measurement this was 27.1 ccm, 13.4 ccm and 3.0 ccm, respectively. The range and absolute and relative standard deviation for these measurements can be found in Table 1. Box-and-whisker plots for the results of the large spheres are shown in Figure 2. The absolute and relative standard deviation in consecutive measurements were the lowest in threshold-based segmentation and consequently higher in all textural-based segmentation algorithms, with the highest values found for the Entropy-based method. In general, it was also found that all three textural-based segmentation algorithms showed on average a larger volume compared to the threshold-based segmentation algorithm.

### 3.2. Patient Measurements

**Figure 3:**
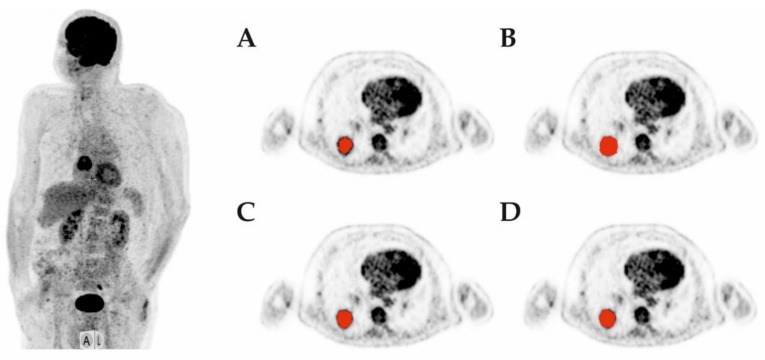
Example of patient 1 (MIP on the left) and segmented volume in transaxial slice for the threshold-based (**A**), the Kurtosis-based (**B**), the Entropy-based (**C**) and the LZE-based (**D**) algorithms.

All four different segmentation methods showed the ability to segment the lung lesion in patient data. The achieved volume was visually reasonable in all cases. Each of the five patients had a single lung lesion located, as shown in Table 2. As in phantom studies, volumes segmented based on textural features have a tendency to be larger than the threshold-based segmented volumes. The largest volumes were obtained by LZE-based segmentation, as already seen for the phantom measurements. An example of a segmented tumor volume can be found in Figure 3, which shows images of the segmented volumes for all four algorithms. Detailed results of the segmented volumes of all five patients as well as the motion vector that was used for correction are shown in Table 2.

For comparison, patient data without motion correction were also used in the different segmentation algorithms, and the results can be found in Table 3. As expected, segmented volumes were larger when PET data were not corrected for motion in all but in two cases (Kurtosis-based segmentation and LZE-based segmentation in patient 4). No relevant differences were found concerning the threshold-based segmented volumes or textural feature-based segmented volumes when comparing motion-corrected data and data not corrected for respiratory movement.

## 4. Discussion

We proposed a novel segmentation algorithm for lung tumors in FDG-PET based on textural features. Based on previous analysis, we chose Kurtosis, Entropy and Long-Zone Emphasis. These textural features, in a study by Jouanjan et al., showed the best ability to differentiate between lung tumor tissue and surrounding lung tissue [24]. In the same study, these parameters were identified to be stable in the time between tracer injection and PET data acquisition, as determined through dual-time-point PET/CT examinations. Many other textural parameters did not provide such as stability in the interval between injection and acquisition and were therefore difficult to implement in algorithms that should be used in clinical routine.

As in the case of lung tumors, respiratory motion is an important source of artifacts in quantification of PET data, especially in target volume estimation [29]. Therefore, we applied the segmentation algorithm on data corrected for respiratory movement by an algorithm previously described by Thomas et al. [34]. However, the proposed algorithm was also applied to non-motion-corrected data for comparison; similar results for the effect of motion correction on the segmented volume were found, as has been previously reported [34]. However, no relevant difference was found between volumes segmented in motion-corrected data and volumes segmented in uncorrected data when using threshold-based algorithms compared to textural-feature-based algorithms.

In phantom studies, we found that segmentation based on Entropy showed the best results with a mean value of 26.5 ccm over 20 measurements amongst the textural-feature-based segmentation algorithms compared to the real sphere volume of 26 ccm. The threshold-based segmentation performed slightly worse with a mean value of 25 ccm. Interestingly, all textural-feature-based methods showed large volumes (mean 27.0 for Kurtosis and 27.1 for LZE). The reason for this may be due to the algorithm itself, as a region only finishes growing when a certain threshold of the textural feature is taken into account. On the other hand, we found that stability between different measurements, in the case of textural features, was worse than in the threshold-based algorithm. The reason for this is that the value of textural features in the grown region is more dependent on the start point than the pure uptake value in a region. Therefore, we found relative standard deviations over 20 measurements for textural-feature-based algorithms ranging between 4.2% and 8.4% compared to values between 1.2% and 5.7% for the threshold-based algorithm. This seems to be a drawback of the presented method, and further steps should be performed to overcome this issue, e.g., by using another definition of the start point rather than manual interaction. One can think of, e.g., the hottest point in a lesion as start point or similar methods to define the start area in a more standardized manner. All segmentation algorithms shared a particular trait: in the case of smaller lesions, variability between different measurements was higher than for larger lesions.

In patient data, we found that the algorithms did provide a sufficient volume in all cases. As for the phantom data, the volumes based on textural feature segmentation were larger than volumes based on threshold-based segmentation (see also Figure 3). However, as no gold standard is available and we included patients scheduled for radiation treatment and the topic is also beyond the scope of this feasibility study, we were unable to determine if the textural parameter-based algorithms were more precise than the threshold-based algorithm in phantom data for Entropy. Our next step will be further studies including the histopathological gold standard as in [38] or [39]. However, these first results about the novel segmentation algorithms are promising, and therefore such studies should be implemented now. Furthermore, in this feasibility study, we focused on phantom data and several first datasets for patients with lung cancer, as this is one of the tumor entities in which PET is often used for radiation treatment. However, further studies should analyze the applicability of the presented algorithms on other tumor entities, such as colorectal carcinoma, breast cancer, brain tumors or osseous or lymph node metastases. In this context, the presented method may be relying on a quite homogeneous background and therefore is not be able to be transferred to all tumor entities easily. Additionally, in the case of lymph nodes, metastases located in the mediastinum or the abdomen, it may be difficult to differentiate between malignant tissue and the background by means of textural features.

Another point taken into account in further investigation is the stability of the proposed algorithms compared to threshold-based algorithms in terms of different scanners, acquisition protocols and reconstruction algorithms. However, this was beyond the scope of this first feasibility study.

A further limitation of the work that needs to be discussed is that the phantom used fillable spheres, so there was hardly any relevant heterogeneity in the activity distribution as would be expected in real tumors. Nevertheless, it seems to be a good model for validation of stability and to see if real lesion size may be reproduced. Additionally, it is important that such a novel algorithm works for both lesions with homogeneous activity distribution as well as inhomogeneous activity distribution if it is to be applied in clinical routines.

## 5. Conclusions

Novel radiomics-based tumor segmentation in FDG-PET has been presented with very good results in phantom studies concerning recovered lesion volume and stability in consecutive measurements. Segmentation based on Entropy seems to be preferable in terms of precision in phantom studies; however, the other algorithms also showed results that are worth following up. Further studies with larger patient cohorts and histopathological standards need to be performed for further validation of the presented algorithms.

## Figures and Tables

**Table 1:** Results of the segmentation using the different algorithms of the phantom data (mean, relative difference to true volume, range, absolute and relative standard deviation).

Segmentation Method	Sphere	Mean (ccm)	Difference (%)	Range (ccm)	Absolute Stdv (ccm)	Relative Stdv (%)
Threshold-based	Large (26 ccm)	25.0	3.9	24.4–25.4	0.3	1.2
	Medium (12 ccm)	10.8	10.0	10.5–11.5	0.4	3.3
	Small (3 ccm)	2.3	23.3	2.0–2.5	0.1	5.7
Kurtosis-based	Large (26 ccm)	27.0	3.9	25.6–30.1	1.2	4.3
	Medium (12 ccm)	13.5	12.5	11.9–15.1	0.8	6.0
	Small (3 ccm)	3.0	0.0	2.5–3.4	0.3	8.2
Entropy-based	Large (26 ccm)	26.5	1.9	21.9–30.4	2.3	8.6
	Medium (12 ccm)	12.2	1.7	10.6–13.4	0.7	5.6
	Small (3 ccm)	3.1	3.3	2.7–3.5	0.2	7.8
LZE-based	Large (26 ccm)	27.1	4.2	25.0–29.0	1.1	4.2
	Medium (12 ccm)	13.4	11.7	12.0–14.9	0.7	4.9
	Small (3 ccm)	3.1	3.3	2.6–3.6	0.3	8.4

**Table 2:** Results of the segmentation using the different segmentation algorithms in five patients after motion correction including motion amplitude in x (anterior–posterior), y (left–right), and z (cranial–caudal) directions.

Patient. No.	Lesion Location	Motion x, y, z (mm)	Segmented Lesion Volume (ccm)			
			thd-Based	Kurtosis-Based	Entropy-Based	LZE-Based
1	Lower-right lobe	2, 1, 12	46.6	50.1	47.6	52.3
2	Lower-left lobe	3, 1, 9	8.2	8.9	9.3	9.0
3	Center-right lobe	2, 2, 8	6.4	12.5	7.9	8.6
4	Lower-right lobe	4, 0, 14	3.1	4.3	3.9	4.6
5	Lower-left lobe	3, 2, 12	32.2	36.7	34.6	34.9

**Table 3:** Results of the segmentation using the different segmentation algorithms in five patients without previous application of the motion-correction algorithm as well as relative difference to the motion-corrected values in parenthesis following the values.

Patient No.	Lesion Location	Segmented Lesion Volume (ccm)			
		thd-Based	Kurtosis-Based	Entropy-Based	LZE-Based
1	Lower-right lobe	56.4 (17.4%)	63.1 (20.6%)	58.6 (18.8%)	64.8 (19.3%)
2	Lower-left lobe	8.7 (5.8%)	9.7 (8.3%)	9.5 (2.1%)	10.1 (10.9%)
3	Center-right lobe	7.1 (9.9%)	14.9 (16.1%)	9.6 (17.7%)	10.2 (15.7%)
4	Right-lower lobe	3.4 (8.8%)	4.3 (0.0%)	5.0 (22.0%)	4.3 (−7.0%)
5	Lower-left lobe	38.0 (15.3%)	40.1 (8.5%)	41.2 (16.0%)	39.6 (11.9%)

## Data Availability

With regard to the German regulations regarding data protection, we cannot make patient data available online or share it. However, all data are available on-site. Due to industrial collaboration and potential patent applications, we cannot make code available.

## Abbreviations

PET	positron emission tomography
NSCLC	non-small-cell lung cancer
KU	Kurtosis
LE	Local Entropy
LZE	Long-Zone Emphasis
FDG	Fluorodeoxyglucose
